# Comparison of Bayesian and frequentist methods for prevalence estimation under misclassification

**DOI:** 10.1186/s12889-020-09177-4

**Published:** 2020-07-20

**Authors:** Matthias Flor, Michael Weiß, Thomas Selhorst, Christine Müller-Graf, Matthias Greiner

**Affiliations:** 1 German Federal Institute for Risk Assessment, Max-Dohrn-Str. 8-10, Berlin, 10589 Germany; 2 University of Veterinary Medicine Hannover, Foundation, Bünteweg 2, Hannover, 30559 Germany

**Keywords:** Prevalence estimation, Imperfect diagnostic test, Misclassification, Bayesian prevalence estimate, Rogan-Gladen estimate, Diagnostic sensitivity, Diagnostic specificity

## Abstract

**Background:**

Various methods exist for statistical inference about a prevalence that consider misclassifications due to an imperfect diagnostic test. However, traditional methods are known to suffer from truncation of the prevalence estimate and the confidence intervals constructed around the point estimate, as well as from under-performance of the confidence intervals’ coverage.

**Methods:**

In this study, we used simulated data sets to validate a Bayesian prevalence estimation method and compare its performance to frequentist methods, i.e. the Rogan-Gladen estimate for prevalence, *RGE*, in combination with several methods of confidence interval construction. Our performance measures are (i) error distribution of the point estimate against the simulated true prevalence and (ii) coverage and length of the confidence interval, or credible interval in the case of the Bayesian method.

**Results:**

Across all data sets, the Bayesian point estimate and the *RGE* produced similar error distributions with slight advantages of the former over the latter. In addition, the Bayesian estimate did not suffer from the *RGE*’s truncation problem at zero or unity. With respect to coverage performance of the confidence and credible intervals, all of the traditional frequentist methods exhibited strong under-coverage, whereas the Bayesian credible interval as well as a newly developed frequentist method by Lang and Reiczigel performed as desired, with the Bayesian method having a very slight advantage in terms of interval length.

**Conclusion:**

The Bayesian prevalence estimation method should be prefered over traditional frequentist methods. An acceptable alternative is to combine the Rogan-Gladen point estimate with the Lang-Reiczigel confidence interval.

## Background

Prevalence estimation is fundamental to a lot of epidemiological studies. However, to obtain an accurate estimation of prevalence, misclassification and measurement errors should be considered as part of bias analysis in epidemiological research [1]. Frequentist and Bayesian methods for bias adjustment of epidemiological risk estimates have been reviewed in Keogh et al. [2] and Shaw et al. [3]. Estimation of prevalence is always based on the application of a diagnostic test to classify samples with respect to the binary trait under investigation. Major sources of uncertainty of prevalence estimates are related to the study design and sampling issues and are usually described using the concepts of bias and precision (statistical parameter uncertainty of the estimate). A typical source of (information) bias is diagnostic misclassification due to imperfect sensitivity and specificity. Rogan and Gladen have derived an estimator for prevalence with adjustment for diagnostic misclassification [4]. This approach requires that unbiased estimates of the diagnostic accuracy be available for the given application (reviewed in [5]). Here, we present a comparison of Bayesian and frequentist methods for prevalence estimation taking into account all relevant uncertainties associated with the study- and meta-data, e.g. the diagnostic test performance.

In the following, *D*^+/−^ denotes the individual disease status and *T*^+/−^ the result of a diagnostic test applied to an individual. The test sensitivity is then defined as the probability that a *D*^+^ individual tests positive, $\widetilde {{Se}\phantom {.}} = \text {Pr}\!\left (T^{+} | D^{+}\right)$, and its specificity is defined as the probability of a *D*^−^ individual testing negative, $\widetilde {{Sp}} = \text {Pr}\!\left (T^{-} | D^{-}\right)$. The disease prevalence, $\widetilde {\pi }$, is the proportion of diseased individuals in a population but can also be thought of as the probability that a randomly sampled individual is diseased. The probability that a test applied to a random individual from such a population yields a positive result is called the apparent prevalence,

1 $$\begin{array}{*{20}l} \widetilde{{AP}} &= \text{Pr}\!\left(T^{+}\right) \notag \\ &= \text{Pr}\!\left(T^{+} | D^{+}\right) \text{Pr}\!\left(D^{+}\right) + \text{Pr}\!\left(T^{+} | D^{-}\right)\text{Pr}\!\left(D^{-}\right) \notag \\ &= \widetilde{{Se}\phantom{.}}\ \widetilde{\pi} + (1 - \widetilde{{Sp}})(1 - \widetilde{\pi}). \end{array} $$

By replacing the true population quantities (denoted by the tilde) in Eq. (1) with estimates of these quantities (denoted by a circumflex accent or “hat”) that are subject to sampling variability and subsequently solving for the prevalence, the Rogan-Gladen point estimate [4] is derived:

2 $$ {RGE} = \widehat{\pi} = \frac{\widehat{{AP}} - \left(1 - \widehat{{Sp}}\right)}{\widehat{{Se}\phantom{.}} - \left(1 - \widehat{{Sp}}\right)}.  $$

In practice, the Rogan-Gladen estimate is truncated to the interval [0, 1] in order to guarantee a proper proportion [6],

3 $$ \left[{RGE}\right]_{0}^{1} = \min\left(\max\left({RGE},\ 0\right),\ 1\right).  $$

The potential need for truncation stems from the fact that for a prevalence estimate to be a valid proportion, 0≤*RGE*≤1, three conditions must be satisfied [4, 6] which may not always be the case in practice:

4a $$\begin{array}{*{20}l} 1 - \widehat{{Sp}} &< \widehat{{Se}\phantom{.}}  \end{array} $$

4b $$\begin{array}{*{20}l} 1 - \widehat{{Sp}} &\leq \widehat{{AP}}  \end{array} $$

4c $$\begin{array}{*{20}l} \widehat{{AP}} &\leq \widehat{{Se}\phantom{.}}  \end{array} $$

Condition (4a), $1 - \widehat {{Sp}} < \widehat {{Se}\phantom {.}}$, should hold for any diagnostic test to meet the basic requirement that a disease be detected better than by chance alone [4]. Failing to satisfy the second condition (4b), $1 - \widehat {{Sp}} \leq \widehat {{AP}}$, results in a negative estimate for the true prevalence, *RGE*<0, whereas violating the third requirement (4c), $\widehat {{AP}} \leq \widehat {{Se}\phantom {.}}$, yields an estimate larger than unity, *RGE*>1 [6]. Note that it is possible for degenerate cases to occur where the first condition is violated but the estimate still yields a value between zero and unity.

In order to quantify the precision of an estimate, it is good practice to accompany the point estimate with a 95% confidence interval (CI). In this study, we included CI construction methods by Clopper and Pearson [7], Sterne [8], Blaker [9], Rogan and Gladen [4], and by Lang and Reiczigel [10].

In a Bayesian framework, credible parameter values are described by probability distributions. In order to provide comparability with the frequentist approach, the mean of a distribution may then be used as a point estimate, and the Bayesian analogue to the CI is the credible interval (CrI) that marks a range of values that combine a specified percentage of the distribution’s probability mass. More specifically, we consider the 95% highest density interval (HDI) which is the shortest of all possible 95% CrI’s.

The aim of this study is to validate a Bayesian model for prevalence estimation with an imperfect diagnostic test and to compare its performance with traditionally used methods. We use simulated data sets based on simulated true parameter values. Our performance measures are (i) estimation error of point estimates and (ii) confidence interval coverage and length. Of special interest is the performance of the Bayesian point estimates in situations when the Rogan-Gladen estimate must be truncated because it otherwise yields a negative value or a value larger than unity.

## Methods

**Fig. 1:**
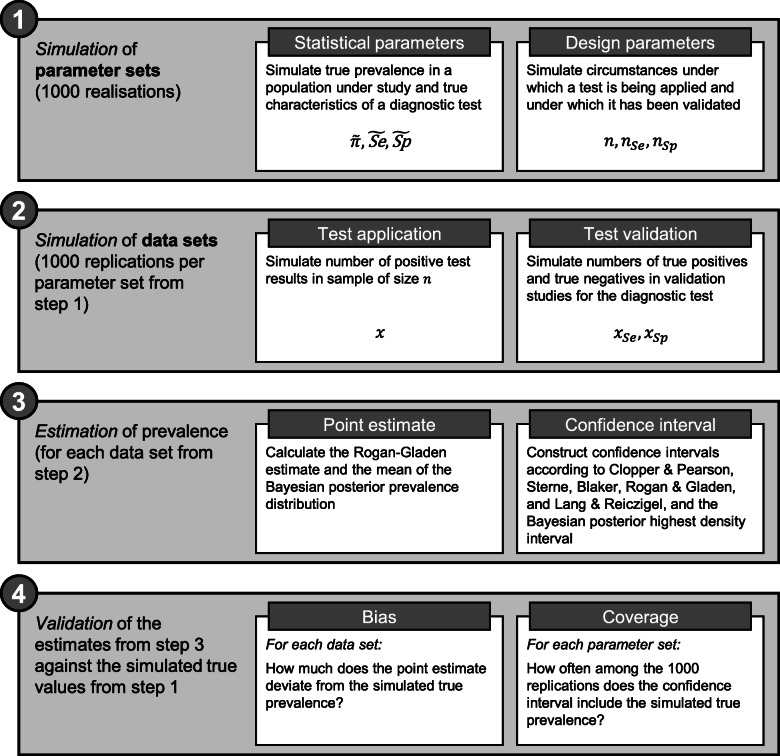
The four steps of the validation study. (1) Simulation of parameter sets to generate true values, (2) simulation of data sets, (3) estimation of prevalence and calculation of confidence intervals, and (4) validation of the estimates against simulated true values

The present validation study consists of four steps: (1) Simulation of parameter sets, including true prevalence values, (2) simulation of data sets, (3) estimation of prevalence for each of the generated data sets, and finally the actual (4) validation of the estimates against the simulated true values. Figure 1 gives an overview of these steps, and we describe each step in detail in the following sections.

All computations were performed on a work station running Ubuntu Linux 18.04.3 LTS, using the statistical software R version 3.6.2 [11] and the MCMC software JAGS version 4.3.0 [12]. The R code used as well es all data sets generated and analysed during this study are available via a Zenodo snapshot, 10.5281/zenodo.3631123.

### Parameter set simulation

We distinguish between statistical parameters and design parameters. The statistical parameters are the true prevalence in a population under study, and the true characteristics of the test that is used to diagnose individuals in a sample drawn from such a population, i.e. true sensitivity and true specificity. The statistical parameters may be estimated by a statistical model. Design parameters describe the circumstances under which data is generated, and are typically chosen by scientists conducting a study or by users applying a diagnostic test. In our case there are three design parameters, all of them sample sizes: The size of the sample that a diagnostic test is applied to, the sample size of a study that has been conducted to validate the sensitivity of the diagnostic test, and likewise the sample size of a validation study of the test’s specificity.

For the purposes of this study we generated 1,000 parameter set realisations consisting of the three statistical and the three design parameters. Simulated true values (indicated by a tilde, $\tilde \ $, in this article) for the statistical parameters, prevalence, sensitivity, and specificity, were randomly drawn from the ranges specified in Table 1. True prevalence $\left (\widetilde {\pi }\right)$ can take on any value between 0% and 100%, and true sensitivity $\left (\widetilde {{Se}\phantom {.}}\right)$ and specificity $\left (\widetilde {{Sp}}\right)$ values are assumed to be at least 60% up to a maximum of 100%.

**Table 1:** Parameter set simulation. For each parameter set realisation, true values for the statistical parameters were simulated by drawing from continuous uniform distributions, $\mathcal {U}(\mathrm {min,\ max})$. Values for the design parameters were simulated by randomly drawing from fixed sets of values (in the case of sample sizes for simulated validation studies of a diagnostic test) or from a discrete uniform distribution (in the case of the sample size for an application of the test)

Parameter	Description	Values sampled from
*Statistical*		
$\quad \quad \widetilde {{Se}\phantom {.}}$	True sensitivity	$\mathcal {U}\left (0.6,\ 1\right)$
$\quad \quad \widetilde {{Sp}}$	True specificity	$\mathcal {U}\left (0.6,\ 1\right)$
$\quad \quad \widetilde {\pi }$	True prevalence	$\mathcal {U}\left (0,\ 1\right)$
*Design*		
*n*_*Se*_	Sample size for a sensitivity validation study	{50,100,200,500,1000,2000, 5000}
*n*_*Sp*_	Sample size for a specificity validation study	{50,100,200,500,1000, 2000, 5000}
*n*	Sample size for a test application	$\mathcal {U}\left (50,\ 2000\right)$

The three design parameters are denoted by *n*_*Se*_, *n*_*Sp*_, and *n*. These enable us to simulate the situations where (i) studies are performed to validate the sensitivity and the specificity of a diagnostic test (with sample sizes *n*_*Se*_ and *n*_*Sp*_, respectively), and situations where (ii) the actual application of a test takes place (namely, the number of individuals that are tested, *n*). The three sample sizes were randomly drawn as specified in Table 1, and exemplary parameter set realisations are shown in Table 2. No correlations were assumed among these parameters.

**Table 2:** Exemplary parameter sets. The first ten out of the total of 1,000 parameter sets

	*Statistical*			*Design*		
Parameter set	$\widetilde {{Se}\phantom {.}}$	$\widetilde {{Sp}}$	$\widetilde {\pi }$	*n*_*Se*_	*n*_*Sp*_	*n*
1	0.7479478	0.9332674	0.4459405	100	200	323
2	0.7768875	0.6758782	0.3946503	50	500	1285
3	0.9816547	0.6470824	0.4837289	500	100	1820
4	0.9410975	0.8721922	0.9188760	1000	100	1524
5	0.8979315	0.6941977	0.8438814	500	200	933
6	0.6628014	0.9243710	0.5173496	5000	1000	427
7	0.9812932	0.9842258	0.4371250	5000	2000	1624
8	0.7668501	0.9065918	0.3431982	1000	2000	642
9	0.8352567	0.6215114	0.0155170	5000	50	1667
10	0.9489196	0.7611306	0.1179912	500	2000	557

### Data set simulation

For each simulated set of parameters we generated 1,000 replications of data sets (replications in the sense that the underlying true values for prevalence, sensitivity, and specificity, are the same for such a set of 1,000 data). Data from diagnostic validation studies were simulated by randomly drawing numbers of true positives, *x*_*Se*_, and numbers of true negatives, *x*_*Sp*_, from the binomial distributions^1^ given in Eq. (5). The number of positive test results, *x*, when applying the test in a population with true prevalence $\widetilde {\pi }$, was simulated analogously:

5 $$\begin{array}{*{20}l} x_{Se} &\sim \mathcal{B}\left(n_{{Se}},\ \widetilde{{Se}\phantom{.}}\right) \notag \\ x_{{Sp}} &\sim \mathcal{B}\left(n_{{Sp}},\ \widetilde{{Sp}}\right) \\ x &\sim \mathcal{B}\left(n, \ \widetilde{{AP}}\right), \notag \end{array} $$

where $\widetilde {{AP}} = \widetilde {{Se}\phantom {.}}\,\widetilde {\pi } + \left (1 - \widetilde {{Sp}}\right)\,\left (1 - \widetilde {\pi }\right)$ may be called the ‘true apparent prevalence’.

Thus for each data set, the maximum likelihood estimators for sensitivity, specificity, and apparent prevalence would be

6 $$\begin{array}{*{20}l} \widehat{{Se}\phantom{.}} &= x_{{Se}} / n_{{Se}} \notag \\ \widehat{{Sp}} &= x_{{Sp}} / n_{{Sp}} \\ \widehat{{AP}} &= x / n \notag \end{array} $$

In our parameter sets, the true values, $\widetilde {{Se}\phantom {.}}$, $\widetilde {{Sp}}$, and $\widetilde {{AP}}$, would (by definition) always meet all of the three conditions (4). However, due to the random sampling procedure described above for simulating (and mimicking random processes occurring in the real world) validation studies and application of a diagnostic test, the maximum likelihood estimators, $\widehat {{Se}\phantom {.}}$, $\widehat {{Sp}\phantom {.}}$, and $\widehat {{AP}}$, will sometimes lead to violation of one or more of the conditions. From the total of 1,000 (parameter set realisations)·1,000 (data set replications)=1,000,000 data sets, we excluded 21 because they failed to meet the first condition, $1 - \widehat {{Sp}} < \widehat {{Se}\phantom {.}}$, arguing that a test that has been validated in this manner would not be applied in practice. The data simulation process thus generated a total of 999,979 data sets. Table 3 shows exemplary data sets from the generation process described in this section.

**Table 3:** Exemplary data sets. The first ten of the replicate data sets for the first parameter set. Statistical and design parameters sampled according to Table 1, data generated according to Eq. (5), and maximum likelihood estimators (MLE) calculated according to Eq. (6)

	*Statistical*			*Design*			*Data*			*MLE*		
Data set	$\widetilde {{Se}\phantom {.}}$	$\widetilde {{Sp}}$	$\widetilde {\pi }$	*n*_*Se*_	*n*_*Sp*_	*n*	*x*_*Se*_	*x*_*Sp*_	*x*	$\widehat {{Se}\phantom {.}}$	$\widehat {{Sp}}$	$\widehat {{AP}}$
1	0.7479478	0.9332674	0.4459405	100	200	323	74	193	129	0.74	0.965	0.3993808
2	0.7479478	0.9332674	0.4459405	100	200	323	70	189	125	0.70	0.945	0.3869969
3	0.7479478	0.9332674	0.4459405	100	200	323	78	189	124	0.78	0.945	0.3839009
4	0.7479478	0.9332674	0.4459405	100	200	323	73	191	109	0.73	0.955	0.3374613
5	0.7479478	0.9332674	0.4459405	100	200	323	76	184	117	0.76	0.920	0.3622291
6	0.7479478	0.9332674	0.4459405	100	200	323	66	188	132	0.66	0.940	0.4086687
7	0.7479478	0.9332674	0.4459405	100	200	323	76	179	121	0.76	0.895	0.3746130
8	0.7479478	0.9332674	0.4459405	100	200	323	75	188	118	0.75	0.940	0.3653251
9	0.7479478	0.9332674	0.4459405	100	200	323	74	187	127	0.74	0.935	0.3931889
10	0.7479478	0.9332674	0.4459405	100	200	323	75	191	127	0.75	0.955	0.3931889

### Estimation of prevalence

In the next step, we estimated prevalence adjusted for misclassification for each of the simulated data sets using frequentist and Bayesian methods.

#### Frequentist estimation

For each data set, we calculated the Rogan-Gladen point estimate for prevalence according to Eq. (3), using the maximum likelihood estimates for the sensitivity and specificity values as well as the apparent prevalence. In order to construct a confidence interval (CI) for the Rogan-Gladen estimate, several different methods are used in the literature. Here, we consider the methods proposed by Clopper and Pearson [7], Sterne [8], Blaker [9], and Rogan and Gladen [4]. All of these methods except for the one by Rogan and Gladen assume that sensitivity and specificity are known, and the MLE values were used in this case. Rogan and Gladen [4] do allow for sensitivity and specificity to be estimated from validation studies (thus taking into account uncertainty around the diagnostic test properties) but due to the normal approximation that they suggest, their CI is known to perform poorly [5]. For a more comprehensive description of how these intervals are constructed we refer to the study by Reiczigel et al. [13]. Indeed, the code we used to calculate these CI’s is based on the code provided in Reiczigel et al. [13] and in Lang and Reiczigel [10].

#### Bayesian estimation

In the Bayesian prevalence estimation model, prior knowledge or belief about the true prevalence as well as the sensitivity and specificity of the diagnostic test is expressed in terms of probability distributions. Prior knowledge in the present context derives from validation studies of the diagnostic test properties but in other situations may also stem from expert opinion. Through data from the application of the test to diagnose samples from a population the model updates the probability distributions which afterwards describe the posterior knowledge about the true prevalence as well as about the sensitivity and specificity of the test.

Posterior probability distributions must often be numerically approximated by random sampling algorithms referred to as Markov Chain Monte Carlo (MCMC) methods. To ensure that values are sampled from a stationary distribution, so-called convergence diagnostics are used.

Our prevalence estimation model was implemented using the JAGS software [12], a Gibbs sampler for MCMC simulations that uses a dialect of the BUGS modeling language [14]. The model, expressed in the BUGS language, is basically a description of the process that provides parameters for data generation:


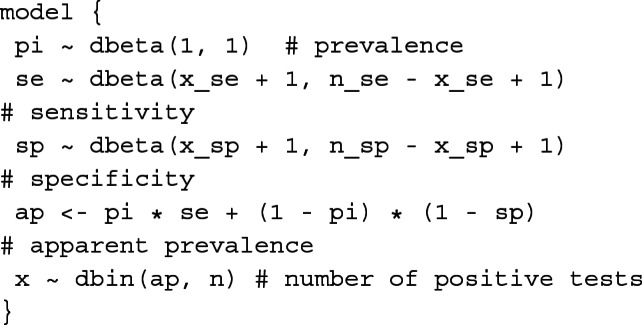


By definition, the variables prevalence, sensitivity, and specificity are all proportions and thus appropriately modeled as *Beta* probability density distributions (dbeta in the BUGS language; the tilde symbol, $\tilde \ $, denotes drawing a random variable from a distribution whereas the left arrow, <-, implies a deterministic relationship). Importantly, this guarantees that all probability mass for each of these variables is restricted to the domain [0, 1] thus eliminating the problem of prevalence estimates that are negative or larger than unity, and the same conveniently holds for limits of credible intervals.

For the prevalence we use the uniform *Beta*(1, 1) as a non-informative prior distribution^2^. In contrast, prior information on the sensitivity and specificity of the diagnostic test is available from their respective validation studies. E.g., if the sensitivity of the test has been validated against a gold standard in a study of size *n*_*Se*_, and *x*_*Se*_ of the truly positive samples yielded a positive test result, then our knowledge about the true sensitivity can be expressed as *Beta*(*x*_*Se*_+1,*n*_*Se*_−*x*_*Se*_+1).

As with all MCMC simulation techniques, using the JAGS software requires that convergence of the Markov chains to a stationary distribution be checked. In order to realize the validation study presented here, i.e. applying our model to approximately 1,000,000 data sets, we made use of the R package runjags [15] which provides an interface to JAGS with capabilities for automated calculation of convergence diagnostics [16] and of appropriate sample length [17] via an autorun function. All chains were initialized explicitly to ensure that convergence can be evaluated appropriately. For all simulations, we used three chains, 1,000 adaptive iterations, a burn-in length of 4,000, and a minimal sample length of 20,000. This yielded an effective sample size of approximately 10,000 on which the prevalence estimates are based. When the Bayesian estimation model was applied to each of the data sets, the Gelman and Rubin’s statistic as used by the autorun function indicated convergence in all cases.

The mean of the posterior distribution –approximated by the three combined chains– is a minimum mean square error estimator and provides a Bayesian point estimate for the true prevalence. The broader the probability distribution, the less certain our knowledge about the true prevalence is. A 95% credible interval (CrI) denotes a range of prevalence estimates that together account for 95% of the probability mass of the distribution. The 95% highest density interval (HDI) is the shortest 95% CrI, such that any value outside the HDI is considered less plausible than the values inside of it. Therefore, the 95% HDI constitutes a natural measure of uncertainty for the estimate. Note that the Bayesian model also provides updated knowledge on the sensitivity and the specificity of the test, but in this study we focus on the performance of the prevalence estimates.

### Validation

In the final validation step of our study, we calculated two performance metrics and their distributions in order to compare the various estimation methods: (i) the estimation error of the point estimates and (ii) the coverage of the confidence or credible intervals.

For each *data set*, the estimation error of the two point estimates (Rogan-Gladen estimate, Bayesian mean) was calculated as the difference to the simulated true value, $\mathrm {estimation\ error} = \widehat {\pi } - \widetilde {\pi }$. Note that a consistent estimator should exhibit a symmetric distribution (across all data sets) of estimation errors that is centered at zero. To investigate which estimator performs better, we carried out a regression of the estimation errors of all Bayesian estimates on the corresponding Rogan-Gladen estimate’s estimation errors. Because obviously there are errors in both estimators, we used a Deming regression and assumed equal error variances for the two distributions. This means that the regression minimizes the sum of squared *orthogonal* distances to the regression line.

For each *parameter set*, coverage of the various frequentist 95% CI’s [4, 7–10] and of the Bayesian 95% HDI was computed as the percentage of the 1,000 replication data sets for which the true prevalence value was contained in the respective interval. Note that this implies assessing the *frequentist* behaviour of the *Bayesian* HDI. A well-behaved 95% confidence (or credible) interval is expected to have a symmetric distribution (across all parameter sets) of coverage values that is centered at its nominal value of 95%. Additionally, we calculated the lengths of the CI’s for all data sets. If one compares two well-behaved CI methods then the one producing shorter intervals is the better one.

## Results

**Fig. 2:**
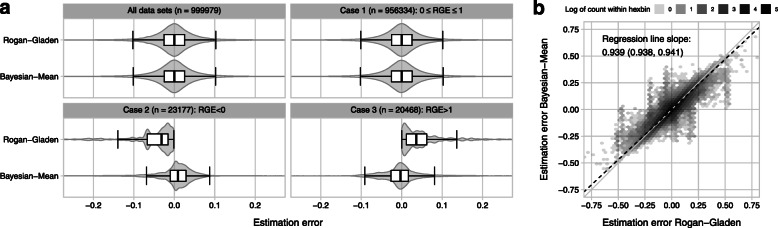
Estimation errors. (**a**) Estimation error distributions of the Rogan-Gladen point estimate and the Bayesian estimate (MCMC mean) across all data sets (top left), and across data sets as classified according to the non-truncated Rogan-Gladen estimate (case 1, top right). The Bayesian estimator shows adequate error distributions for the data sets with a truncated *RGE* (cases 2 and 3, bottom row). (**b**) Comparison of the estimation errors of the Bayesian mean and the Rogan-Gladen estimate for all data sets. Hexagonal binning is used to deal with overplotting, and the hex gray scale codes for the number of data sets that fall within it. The dashed black line shows a Deming regression of the Bayesian estimation error on the Rogan-Gladen estimation error. Its slope is 0.939 with a confidence interval of (0.938, 0.941)

In general, the Rogan-Gladen point estimate as well as the Bayesian estimator (i.e. the MCMC mean) yield a consistent point estimate for the prevalence. More specifically, estimation error distributions of the two methods look very similar when compared across the entirety of all simulated data sets (Fig. 2a, top left). This property of being consistent also holds true for data subsets in which the Rogan-Gladen estimate yields a value between zero and unity (Fig. 2a, top right). However, for subsets of the data in which the non-truncated Rogan-Gladen estimate is negative (Fig. 2a, bottom left) or larger than unity (Fig. 2a, bottom right), the Bayesian estimator is more consistent and exhibits a distribution approximately symmetrical to an estimation error of zero. Its asymmetrical error distribution indicates a deficiency of the Rogan-Gladen estimator.

In order to compare the two estimators in more detail, we plotted their estimation error values against one another for all of the 999,979 data sets (see Fig. 2b). To deal with overplotting, we used hexagonal binning; the darker the shade of a hexbin the more data sets were registered within that bin. In general, the hexbins are distributed along the diagonal showing that the estimation errors of the two estimators behave similarly. The correlation coefficient for the two estimation errors is *r*=0.921, and the orthogonal regression has a slope of 0.939. The confidence interval for the slope estimate, (0.938, 0.941), does not include the diagnonal (slope 1) thus revealing that overall the Bayesian estimator performs slightly better than the Rogan-Gladen estimate. A linear regression analysis for both estimators suggests that the residual estimation error after adjusting prevalence estimates for diagnostic sensitivity and specificity does depend on the application scenario as represented by our design variables for sample sizes, true prevalence, true sensitivity and true specificity, but that the effects are only very minor for the whole model, the individual parameters, as well as their first-order interactions (see Additional file 1).

**Fig. 3:**
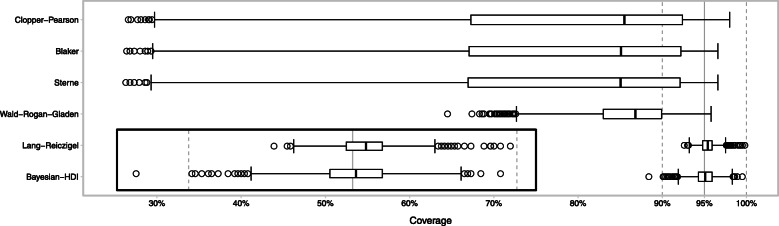
Confidence interval coverage. Coverage by parameter set for several methods of 95*%* confidence interval (CI) construction. The nominal coverage of 95*%* is marked by a solid gray line, and the dahed lines mark coverage values of 90*%* and 100*%*. The traditional CI’s (Clopper-Pearson, Blaker, Sterne, and Wald-Rogan-Gladen) all exhibit significant under coverage. The bottom left inset shows the Lang-Reiczigel CI’s and the Bayesian HDI’s coverage distributions in more detail. It reveals the Lang-Reiczigel CI tends to have some over coverage and the Bayesian HDI’s coverage appears to be more symmetrical around the nominal 95*%* value

The coverage of the Bayesian HDI credible intervals and of the Lang-Reiczigel CI is in good accordance with their nominal value of 95%, as can be seen in Fig. 3. This is in stark contrast to traditional confidence intervals which exhibit coverage much lower than 95%. In fact, the nominal 95% are not even included in the interquartile ranges of coverage values for these traditional methods.

**Fig. 4:**
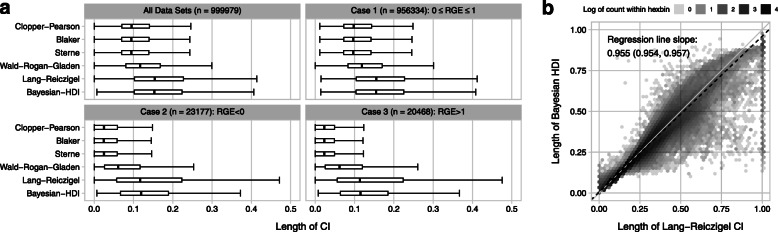
Confidence interval length. (**a**) Confidence (credible) interval length for several methods across all data sets (top left), and across data sets as classified according to the non-truncated Rogan-Gladen estimate. Traditional CI’s are generally shorter than the Lang-Reiczigel CI and the Bayesian HDI. (**b**) Comparison of the lengths of the Bayesian HDI and the Lang-Reiczigel CI for all data sets. Hexagonal binning is used to deal with overplotting, and the hex gray scale codes for the number of data sets that fall within it. A Deming regression is shown as a dashed black line, its slope is 0.955 with a confidence interval of (0.954, 0.957)

Coverage is one performance metric for a confidence (or credible) interval, length of the interval is the other. Figure 4a shows that traditional CI’s (Clopper-Pearson, Blaker, Sterne, Wald-Rogan-Gladen) are much shorter than the Lang-Reiczigel CI and the Bayesian HDI, the two of which have very similar lengths. This pattern is even more pronounced for the data subsets in which the Rogan-Gladen estimate has to be truncated at zero (case 2; Fig. 4a bottom left) or at unity (case 3; Fig. 4a bottom right).

Figure 4b shows how the lengths of the Bayesian HDI and of the best performing (in terms of coverage) conventional confidence interval, the Lang-Reiczigel CI, relate to one another. The correlation coefficient is *r*=0.975. The dashed black line shows an orthogonal Deming regression, again under the assumption that both interval lengths have equal variance. The slope of the regression is 0.955 (0.954, 0.957) showing that the Bayesian HDI tends to be slightly narrower than the Lang-Reiczigel CI.

## Discussion

In this simulation study we evaluated the validity of a Bayesian method to estimate true prevalence based on the results of imperfect diagnostic tests. The Bayesian point estimate of the true prevalence performed slightly better than the conventional Rogan-Gladen estimate. Our study demonstrated that the traditional confidence intervals, Clopper-Pearson, Blaker, Sterne, and Wald-Rogan-Gladen exhibit considerable under-coverage and should be considered unfit for prevalence estimation under misclassification. In contrast, both the Lang-Reiczigel CI and the Bayesian HDI can be considered fit for use as they provide coverage close to the level of 95*%*. The fact that the traditional methods generally provide much narrower CI’s than the Lang-Reiczigel CI or the Bayesian HDI does obviously not ameliorate this shortcoming but rather gives an impression of certainty that is unwarranted. It could be said that the Lang-Reiczigel CI even errs slightly to the safe side (see inset in Fig. 3).

A basic assumption underlying the present study is that the sensitivity and specificity of the diagnostic test have been validated in a manner appropriate for the given application of the test. This refers to the concept of “operational parameters” and requires that the panel selection (of truly positives and truly negatives) for the validation of sensitivity and specificity of the test is representative for its application in the field. This assumption, however, needs to be made independent of whether one uses the conventional Rogan-Gladen estimate or a Bayesian method.

Violations of the conditions (4b) or (4c) can indicate that the prior information about test accuracy is inconsistent with the application data. In this case it may be a better option to refrain from adjustment for misclassification until reliability and relevance of the diagnostic’s parameters are clarified.

Our simulations resulted in calculated Rogan-Gladen estimates outside the possible range of [0, 1] in 4% of the cases. This percentage results from our choices of simulation scenarios and cannot be extrapolated to real applications. However, bias adjustment has led to “impossible” results which does occurs in practice. E.g., Moujaber et al. [18] compared prevalences of *Heliobacter pylori* infection in Australia among different age groups but only reported apparent prevalences. Had they corrected for sensitivity and specificity of the ELISA test used, prevalence estimates for two age groups would have been negative. This becomes obvious also in other statistical models, for example when bias adjustment results in negative count data. Lash et al. [1] noted that encountering impossible adjusted data is often of substantive importance, as it may represent a fundamental disconnect between the priors and the data or data model, and may signal poor prior information, poor data modelling or unrecognized data problems. We believe that this reflects well the situation of truncated Rogan-Gladen estimates. As pointed out elsewhere [5], an adjustment for sensitivity and specificity can be very misleading if those accuracy parameters are not valid for the given prevalence study.

For the construction of their confidence interval, Lang and Reiczigel –as we did in our study– use non-informative *Beta*(1, 1) priors for sensitivity and specificity and update the probability distributions according to the results of the validation studies and thus gain increased performance by adopting Bayesian concepts. An advantage of our model may be seen in the opportunity to easily incorporate expert judgment on sensitivity and specificity instead of validation studies. This might be particularly useful in situations where a lot of practical experience is available but the existence of a reliable gold standard is questionable.

Latent class models for estimating sensitivity and specificity without gold standard [19] are related to our approach since in this context, prevalence estimation adjusted for sensitivity and specificity occurs as a “by-product” of estimating accuracy parameters. Only four out of 64 empirical studies on human populations reported prevalence estimation as primary goal of latent class modelling [20].

There is a recent tendency in health risk assessment studies to replace the traditional Monte Carlo methods by Bayesian network analysis [21]. The Bayesian method for the estimation of true prevalence validated in our study can easily be incorporated into a Bayesian network analysis by using the BUGS laguage (e.g. by using JAGS) and thus provides a single software solution for the complex task of risk assessment in scenarios with a high level of uncertainty.

Even small improvements in the precision of prevalence estimations are likely to contribute considerably to ameliorate the quality of risk analysis studies because in these studies information about prevalence is incorporated into larger systems of dependencies such that biases display a tendency to be enlarged in the process of end point estimation.

## Conclusions

Prevalence estimations can easily be adjusted for diagnostic misclassification if the diagnostic performance of the test or instrument has been characterized in terms of sensitivity and specificity in a validation study. Furthermore, the use of a Bayesian model is a flexible approach for quantifying the combined uncertainties of all model parameters as it can be informed by empirical data as well as by expert opinion. In a validation study on simulated data sets, Bayesian estimates of the true prevalence and the inherent uncertainty proved superior to traditional frequentist methods, exhibiting less bias than the Rogan-Gladen estimate and better coverage than conventional methods.

## Supplementary information

**Additional file 1** Linear regression analysis. Regression analysis using linear models with the prevalence point estimate’s estimation error as dependent variable and all z-transformed statistical and design variables $\left(\widetilde{\pi}, \widetilde{Se}, \widetilde{Sp}, {n}, n_{Se}, n_{Sp}\right)$ as well as first-order interactions as independent variables, conducted for both the Rogan-Gladen estimator and the Bayesian estimator.

## Data Availability

The R code used in this study is available on GitHub, https://github.com/BfRstats/bayespem-validation-code. From this repository, a Zenodo snapshot was created that includes the code as well es all data sets generated and analysed during this study, 10.5281/zenodo.3631123.

## Abbreviations

AP: Apparent prevalence
B: Binomial distribution
BUGS: Bayesian inference using Gibbs sampling
language for specifying Bayesian models; CI: Confidence interval
CrI: Credible interval
*D*^+/−^: Disease status
HDI: Highest density interval
JAGS: Just another Gibbs sampler
software for simulation of Bayesian models using MCMC; MCMC: Markov chain Monte Carlo
MLE: Maximum likelihood estimator
*π*: Prevalence
Pr: Probability
RGE; Rogan-Gladen estimator for prevalence; Se: Sensitivity
Sp: Specificity
*T*^+/−^: Test result

## Footnotes

1 The binomial distribution $\mathcal {B}(n,\ p)$ is the discrete probability distribution of the number of successes in a series of independent trials where parameters *n* and *p* are the number of trials and the success probability for each trial, respectively.

2 More accurately, the *B**e**t**a*(1, 1) prior is *weakly* informative, as it implicitly considers all possible values to be equally likely. However, for the sake of simplicity we will use the term *non-informative* throughout this article.
