# Supplementary material for: Comparison of Bayesian and frequentist methods for prevalence estimation under misclassification
Source: BMC Public Health. 2020 Jul 20;20:1135. doi: 10.1186/s12889-020-09177-4 (PMC7370479; doi:10.1186/s12889-020-09177-4)
Supplement: Supplementary file 1 — Additional file 1 Linear regression analysis. Regression analysis using linear models with the prevalence point estimate’s estimation error as dependent variable and all z-transformed statistical and design variables $\left(\widetilde{\pi}, \widetilde{Se}, \widetilde{Sp}, {n}, n_{Se}, n_{Sp}\right)$π~,Se~,Sp~,n,nSe,nSp as well as first-order interactions as independent variables, conducted for both the Rogan-Gladen estimator and the Bayesian estimator. [file 12889_2020_9177_MOESM1_ESM.html]

Linear Regression Analysis


Code 

- Show All Code
- Hide All Code

# Linear Regression Analysis

# Summary

We conducted a regression analysis in which we have used linear models with the prevalence point estimate’s estimation error as dependent variable and allz-transformed statistical and design variables (see Section Definitions and Preparations) as well as first-order interactions as independent variables.
The sections below present details on the regression coefficients and residual plots both for the Rogan-Gladen estimator (Section Rogan-Gladen) and the Bayesian estimator (Section Bayesian Mean) as well as a tornado plot comparing the sensitivity of the two estimator’s errors on the statistical and design variables (Section Comparison of the Estimators).

The results suggest that the residual estimation error after adjusting prevalence estimates for diagnostic sensitivity and specificity does depend on the application scenario as represented by our design variables for samples sizes, true prevalence, true sensitivity and true specificity.
However, the results show only very minor effects for the whole model and for the individual parameters as indicated by very small adjusted R-squared values and small linear regression coefficients for both the frequentist and the Bayesian methods.

E.g., the strongest effect in our simulated data is exercised by the true prevalence on the Bayesian estimator.
However, with an estimated regression coefficient of -0.008334 this means that if the true prevalence increases by, say, two standard deviation (for our simulated data with a uniform true prevalence distribution, this is equivalent to an increase by 0.58 on the original scale), the Bayesian estimator’s error decreases by 0.0167 on the original scale.

Note that, interestingly, the sample size of the test application bears almost no influence on the estimation error at all, neither for the Bayesian nor the frequentist method.

The residual plots show that for both estimators, the estimation error’s spread decreases with increasing true sensitivity and true specificity.
The distribution of residuals for the true prevalence, on the other hand, is more asymmetric towards the lower as well as the upper end, reflecting the prevalence bounds of zero and one, respectively.
E.g., for a very low true prevalence the estimation error could take large positive values but only small negative ones.

# Definitions and Preparations

Design parameters:

- n: Sample size for a test application
- n\_se: Sample size for a sensitivity validation study
- n\_sp: Sample size for a specificity validation study

Statistical parameters:

- pi\_true: True prevalence
- se\_true: True sensitivity
- sp\_true: True specificity

**All parameters were z-transformed prior to the regression analysis.**

Estimation errors:

- error\_RG: Estimation error of the Rogan-Gladen estimator
- error\_BM: Estimation error of the Bayesian estimator (MCMC mean)

The following data frame shows the z-transformed parameters and estimation errors for the first ten data sets.

```
scale_this <- function(x){
    (x - mean(x, na.rm = TRUE)) / sd(x, na.rm = TRUE)
}
dat <- bias_df %>% 
    left_join(datasets, by = "data_id") %>% 
    select(data_id, par_id,
           pi_true, se_true, sp_true, 
           n, n_se, n_sp, 
           `Bayesian-Mean`, `Rogan-Gladen`) %>% 
    mutate(pi_true = scale_this(pi_true),
           se_true = scale_this(se_true),
           sp_true = scale_this(sp_true),
           n       = scale_this(n),
           n_se    = scale_this(n_se),
           n_sp    = scale_this(n_sp)) %>%
    rename(error_BM = `Bayesian-Mean`,
           error_RG = `Rogan-Gladen`) %>% 
    print()
```

```
## # A tibble: 999,979 x 10
##    data_id par_id pi_true se_true sp_true     n   n_se   n_sp  error_BM error_RG
##      <int>  <int>   <dbl>   <dbl>   <dbl> <dbl>  <dbl>  <dbl>     <dbl>    <dbl>
##  1       1      1  -0.159  -0.474    1.19 -1.31 -0.723 -0.664  0.0758    0.0709 
##  2       2      1  -0.159  -0.474    1.19 -1.31 -0.723 -0.664  0.0743    0.0688 
##  3       3      1  -0.159  -0.474    1.19 -1.31 -0.723 -0.664  0.0112    0.00772
##  4       4      1  -0.159  -0.474    1.19 -1.31 -0.723 -0.664 -0.0159   -0.0190 
##  5       5      1  -0.159  -0.474    1.19 -1.31 -0.723 -0.664 -0.0274   -0.0309 
##  6       6      1  -0.159  -0.474    1.19 -1.31 -0.723 -0.664  0.143     0.135  
##  7       7      1  -0.159  -0.474    1.19 -1.31 -0.723 -0.664 -0.0308   -0.0343 
##  8       8      1  -0.159  -0.474    1.19 -1.31 -0.723 -0.664  0.000459 -0.00344
##  9       9      1  -0.159  -0.474    1.19 -1.31 -0.723 -0.664  0.0448    0.0403 
## 10      10      1  -0.159  -0.474    1.19 -1.31 -0.723 -0.664  0.0523    0.0479 
## # ... with 999,969 more rows
```

---

# Rogan-Gladen

```
fit_RG <- lm(
    error_RG ~ (pi_true + se_true + sp_true + n + n_se + n_sp)^2,
    data = dat
)
stargazer(fit_RG, type = "html",
          title = "Table S1. Regression coefficients for the Rogan-Gladen estimation error.",
          dep.var.caption = "",
          ci = TRUE, single.row = TRUE,
          star.cutoffs = c(0.05, 0.01, 0.001),
          notes = "<em>&#42;&nbsp;p&nbsp;&lt;&nbsp;0.05;&nbsp;&#42;&#42;&nbsp;p&nbsp;&lt;&nbsp;0.01;&nbsp;&#42;&#42;&#42;&nbsp;p&nbsp;&lt;&nbsp;0.001</em>", 
          notes.append = FALSE,
          notes.align = "c",
          digits = 4, digits.extra = 6)
```

**Table S1. Regression coefficients for the Rogan-Gladen estimation error.**

|  | |
|  | error\_RG |
|  | |
| pi\_true | -0.0023\*\*\* (-0.0024, -0.0022) |
| se\_true | -0.00004 (-0.0002, 0.0001) |
| sp\_true | 0.0003\*\*\* (0.0001, 0.0004) |
| n | -0.0001 (-0.0002, 0.00003) |
| n\_se | -0.0003\*\*\* (-0.0005, -0.0002) |
| n\_sp | 0.0004\*\*\* (0.0003, 0.0005) |
| pi\_true:se\_true | 0.0017\*\*\* (0.0016, 0.0018) |
| pi\_true:sp\_true | 0.0019\*\*\* (0.0018, 0.0020) |
| pi\_true:n | 0.0007\*\*\* (0.0006, 0.0008) |
| pi\_true:n\_se | 0.0007\*\*\* (0.0006, 0.0009) |
| pi\_true:n\_sp | 0.0007\*\*\* (0.0005, 0.0008) |
| se\_true:sp\_true | -0.0001 (-0.0002, 0.00002) |
| se\_true:n | 0.0001\* (0.000004, 0.0002) |
| se\_true:n\_se | 0.0002\*\* (0.00004, 0.0003) |
| se\_true:n\_sp | 0.0001 (-0.00005, 0.0002) |
| sp\_true:n | -0.00005 (-0.0002, 0.0001) |
| sp\_true:n\_se | -0.00004 (-0.0002, 0.0001) |
| sp\_true:n\_sp | -0.0001 (-0.0002, 0.00003) |
| n:n\_se | 0.0003\*\*\* (0.0002, 0.0004) |
| n:n\_sp | 0.0002\*\*\* (0.0001, 0.0003) |
| n\_se:n\_sp | -0.0001 (-0.0002, 0.0001) |
| Constant | 0.0001 (-0.00002, 0.0002) |
|  | |
| Observations | 999,979 |
| R2 | 0.0044 |
| Adjusted R2 | 0.0044 |
| Residual Std. Error | 0.0572 (df = 999957) |
| F Statistic | 209.6787\*\*\* (df = 21; 999957) |
|  | |
| *Note:* | *\* p < 0.05; \*\* p < 0.01; \*\*\* p < 0.001* |

---

```
resid_panel(fit_RG, plots = "all")
```

Figure S1: Diagnostic residual plots for the Rogan-Gladen estimation error.

---

```
resid_xpanel(fit_RG, title.opt = FALSE)
```

Figure S2: Plots of residuals vs parameters for the Rogan-Gladen estimation error.

---

```
tidy_RG <- tidy(fit_RG) %>% 
    mutate(estimator = "Rogan-Gladen") %>% 
    filter(term != "(Intercept)")
tidy_RG %>% 
    ggplot(aes(x = reorder(term, -estimate), y = estimate)) +
    geom_col(width = 0.5, fill = "#969696", orientation = "x") +
    scale_x_discrete(labels = function(l) parse(text=l)) +
    scale_y_continuous("Estimate of linear regression coefficient", 
                       breaks = scales::pretty_breaks(n = 7)) +
    coord_flip() +
    theme_bw(base_size = 12) +
    theme(axis.title.x = element_text(margin = margin(1, 0, 0, 0, "lines")),
          axis.title.y = element_blank(),
          legend.key.width = unit(1.5, "lines"),
          legend.key.height = unit(0.3, "lines")) +
    panel_border()
```

Figure S3: Tornado plot for the Rogan-Gladen estimation error.

---

# Bayesian Mean

```
fit_BM <- lm(
    error_BM ~ (pi_true + se_true + sp_true + n + n_se + n_sp)^2,
    data = dat
)
stargazer(fit_BM, type = "html",
          title = "Table S2. Regression coefficients for the Bayesian estimation error.",
          dep.var.caption = "",
          ci = TRUE, single.row = TRUE,
          star.cutoffs = c(0.05, 0.01, 0.001),
          notes = "<em>&#42;&nbsp;p&nbsp;&lt;&nbsp;0.05;&nbsp;&#42;&#42;&nbsp;p&nbsp;&lt;&nbsp;0.01;&nbsp;&#42;&#42;&#42;&nbsp;p&nbsp;&lt;&nbsp;0.001</em>",
          notes.append = FALSE,
          notes.align = "c",
          digits = 4, digits.extra = 6)
```

**Table S2. Regression coefficients for the Bayesian estimation error.**

|  | |
|  | error\_BM |
|  | |
| pi\_true | -0.0083\*\*\* (-0.0084, -0.0082) |
| se\_true | 0.0004\*\*\* (0.0003, 0.0005) |
| sp\_true | -0.0005\*\*\* (-0.0006, -0.0004) |
| n | 0.0001 (-0.00005, 0.0002) |
| n\_se | -0.0019\*\*\* (-0.0020, -0.0018) |
| n\_sp | 0.0024\*\*\* (0.0023, 0.0025) |
| pi\_true:se\_true | 0.0068\*\*\* (0.0067, 0.0069) |
| pi\_true:sp\_true | 0.0070\*\*\* (0.0069, 0.0071) |
| pi\_true:n | 0.0034\*\*\* (0.0033, 0.0035) |
| pi\_true:n\_se | 0.0022\*\*\* (0.0021, 0.0023) |
| pi\_true:n\_sp | 0.0021\*\*\* (0.0020, 0.0022) |
| se\_true:sp\_true | 0.0004\*\*\* (0.0003, 0.0005) |
| se\_true:n | 0.0005\*\*\* (0.0004, 0.0006) |
| se\_true:n\_se | -0.0002\*\*\* (-0.0003, -0.0001) |
| se\_true:n\_sp | -0.0001 (-0.0002, 0.0001) |
| sp\_true:n | -0.0001 (-0.0002, 0.00005) |
| sp\_true:n\_se | 0.000003 (-0.0001, 0.0001) |
| sp\_true:n\_sp | 0.0007\*\*\* (0.0006, 0.0008) |
| n:n\_se | 0.0001 (-0.00004, 0.0002) |
| n:n\_sp | 0.0005\*\*\* (0.0004, 0.0006) |
| n\_se:n\_sp | -0.0002\*\*\* (-0.0003, -0.0001) |
| Constant | 0.0005\*\*\* (0.0004, 0.0006) |
|  | |
| Observations | 999,979 |
| R2 | 0.0693 |
| Adjusted R2 | 0.0692 |
| Residual Std. Error | 0.0522 (df = 999957) |
| F Statistic | 3,543.1950\*\*\* (df = 21; 999957) |
|  | |
| *Note:* | *\* p < 0.05; \*\* p < 0.01; \*\*\* p < 0.001* |

---

```
resid_panel(fit_BM, plots = "all")
```

Figure S4: Diagnostic residual plots for the Bayesian estimation error.

---

```
resid_xpanel(fit_BM, title.opt = FALSE)
```

Figure S5: Plots of residuals vs parameters for the Bayesian estimation error.

---

```
tidy_BM <- tidy(fit_BM) %>% 
    mutate(estimator = "Bayesian-Mean") %>% 
    filter(term != "(Intercept)") 
tidy_BM %>% 
    ggplot(aes(x = reorder(term, -estimate), y = estimate)) +
    geom_col(width = 0.5, fill = "#d9d9d9", orientation = "x") +
    scale_x_discrete(labels = function(l) parse(text=l)) +
    scale_y_continuous("Estimate of linear regression coefficient", 
                       breaks = scales::pretty_breaks(n = 7)) +
    coord_flip() +
    theme_bw(base_size = 12) +
    theme(axis.title.x = element_text(margin = margin(1, 0, 0, 0, "lines")),
          axis.title.y = element_blank(),
          legend.key.width = unit(1.5, "lines"),
          legend.key.height = unit(0.3, "lines")) +
    panel_border()
```

Figure S6: Tornado plot for the Bayesian estimation error.

---

# Comparison of the Estimators

```
bind_rows(tidy_BM, tidy_RG) %>%
    mutate(estimator = factor(estimator, 
                              levels = c("Bayesian-Mean", "Rogan-Gladen"),
                              ordered = TRUE)) %>% 
    mutate(abs_est = abs(estimate),
           sign_est = sign(estimate)) %>%
    group_by(term) %>%
    mutate(max_abs = max(abs_est),
           is_max = as.integer(abs_est == max_abs),
           max  = is_max * sign_est * max_abs) %>%
    ungroup() %>%
    arrange(max, term) %>%
    ggplot(aes(x = reorder(term, -max), y = estimate, 
               fill = estimator)) +
    geom_col(width = 0.8, position = "dodge") +
    scale_x_discrete(labels = function(l) parse(text=l)) +
    scale_y_continuous("Estimate of linear regression coefficient", 
                       breaks = scales::pretty_breaks(n = 7)) +
    scale_fill_manual("", values = c("#d9d9d9", "#969696"),
                      guide = guide_legend(reverse = TRUE)) +
    coord_flip() +
    theme_bw(base_size = 12) +
    theme(axis.title.x = element_text(margin = margin(1, 0, 0, 0, "lines")),
          axis.title.y = element_blank(),
          legend.key.width = unit(1.5, "lines"),
          legend.key.height = unit(0.3, "lines")) +
    panel_border()
```

Figure S7: Tornado plot comparing the Rogan-Gladen and the Bayesian estimation errors.
